# A proactive approach to prevent non-communicable diseases through screening and educating emergency department attendees to adopt healthy lifestyles: Study protocol for a pragmatic, multicenter, randomized controlled trial

**DOI:** 10.1371/journal.pone.0327558

**Published:** 2025-07-03

**Authors:** William Ho Cheung Li, Eliza Lai-Yi Wong, Wei Xia, Hong Chen, Sik-Hon Tsui, Yiu Cheung Chan, Kai Yeung Cheung, Yuen Fan Leung, Long Kwan Laurie Ho, Kai Chow Choi, Oi-kwan Joyce Chung

**Affiliations:** 1 The Nethersole School of Nursing, The Chinese University of Hong Kong, Hong Kong, China; 2 JC School of Public Health and Primary Care, The Chinese University of Hong Kong, Hong Kong, China; 3 School of Nursing, Sun Yat-Sen University, Guangzhou, China; 4 Accident and Emergency Department, Queen Mary Hospital, Hong Kong, China; 5 Accident and Emergency Department, United Christian Hospital, Hong Kong, China; 6 Accident and Emergency Department, Kwong Wah Hospital, Hong Kong, China; 7 School of Nursing, The Hong Kong Polytechnic University, Hong Kong, China; PLOS: Public Library of Science, UNITED KINGDOM OF GREAT BRITAIN AND NORTHERN IRELAND

## Abstract

Noncommunicable diseases (NCDs) have become the leading contributors to morbidity and mortality worldwide, responsible for 74% of all deaths. The major risk factors that substantially contribute to and significantly increase the risk of dying from NCDs include tobacco and alcohol use, unhealthy diet, and physical inactivity. Proactive prevention strategies are vital in reducing the burden. Presenting at the emergency department (ED) can be an excellent “teachable moment” to intervene for unhealthy behaviors because people seeking medical treatment from doctors at EDs may be more motivated to adopt healthy lifestyles. We aim to examine the effectiveness of a general health promotion intervention based on self-determination theory in helping ED attendees adopt a healthy lifestyle. A randomized clinical trial will be conducted on Chinese adults aged ≥18 years attending the EDs of five major acute care hospitals in Hong Kong. Participants will be randomized 1:1 into intervention and control groups (n = 586 per group). Intervention group will receive a brief telephone intervention using the AWARD (Ask, Warn, Advise, Refer and Do-it-again) model, weekly personalized instant messages and four 1-minute videos focused on the desired behaviors via WeChat/WhatsApp, and follow-up assessments of behavior changes at 3, 6, and 12 months. While control group will receive similar brief intervention which only advises them to adopt a healthy lifestyle, similar number of SMS messages containing only general health advice, and follow-up assessments at same schedule with the intervention group. Outcome measures include the composite event rate of adopting at least one of the four healthy lifestyles at 6 (primary outcome) and 12 months measured by a behavioral risk-factor questionnaire and improvement in health-related quality of life at 6 and 12 months measured by the EuroQoL 5-Dimension 5-level (EQ-5D-5L) questionnaire. Ethical approval has been obtained. This trial is registered at ClinicalTrials.gov on March 17, 2025: NCT06889792.

## Introduction

Noncommunicable diseases (NCDs) have been the primary contributors to global morbidity and mortality, accounting for 74% of all deaths worldwide [1,2]. The leading causes of NCDs mortality are cardiovascular diseases, cancers, respiratory diseases, and diabetes, accounting for over 80% of all premature NCD deaths [1]. Modifiable risk factors such as tobacco and alcohol use, physical inactivity, and unhealthy diets substantially contribute to and significantly increase the risk of dying from NCDs [3]. Implementing prevention strategies by promoting healthy lifestyles is crucial to mitigate the burden of NCDs [4,5].

Many interventional studies targeting health-risk behaviours, such as tobacco use or excessive alcohol use, have been found effective [6,7], however few studies targeted changing multiple health-risk behaviours. Several systematic reviews and meta-analyses have been conducted to examine the interventions for multiple health-risk behaviours and most are education about health-risk behaviours and skills or behaviour counselling to adopt healthy lifestyles [8–12]. However, these systematic reviews reported inconsistent findings and methodological flaws, such as small sample sizes in many included studies, a lack of theory-driven interventions, and failure to evaluate long-term outcomes [8–13]. Methodological heterogeneity, including study design, measurement tools, study populations, seriously affect the quality of the evidence and limit the generalizability of their findings [8–12]. In addition, the sustainable effects of proposed interventions and their cost-effectiveness are rarely explored. Most importantly, previous studies on health-risk behaviours and interventions were mostly conducted in Western countries, with limited research conducted among Chinese ethnic groups [8–12].

Hong Kong is facing a growing burden of NCDs, which was exacerbated by an aging population [14,15]. In 2021, a large population survey (N = 5,737) investigating the health-risk behaviours of Chinese adults in Hong Kong showed that 80.3% had at least one and 47.0% had two or more health-risk behaviours [6,16]. In response to the challenges posed by the increasing prevalence of NCDs, the government has committed to enhancing district-based primary healthcare services by establishing health centres in all districts across Hong Kong and launching the Chronic Disease Co-Care Pilot Scheme in 2023 [17]. However, many Chinese adults lack motivation or experience difficulty in adopting a healthy lifestyle, especially if they do not receive advice or support from healthcare professionals [18–21]. The receipt of medical attention at an ED by a person in physical discomfort can serve as an excellent ‘teachable moment’ because it provides an invaluable opportunity to initiate a healthy lifestyle. People who consult a doctor in an ED are more likely to modify their risk behaviours to improve their health. A proactive approach is required to screen people with health-risk behaviours and help them adopt healthy lifestyles, including quitting smoking, avoiding excessive alcohol consumption, maintaining a balanced diet, and engaging in regular physical activity.

Previous studies have shown that individuals with a general intention to improve their health are more likely than others to engage in a desirable healthy lifestyle, and once engaged, will progress to the adopting of another healthy lifestyle [22,23]. Based on this concept and results, we conducted a pilot study to evaluate the effectiveness of a general health promotion strategy using instant messaging platform (WhatsApp or WeChat) in helping smokers with NCDs quit smoking [24]. The study revealed that the intervention group had a higher biochemically validated abstinence rate than the control group at 12 months (16.7% [5 of 30] vs. 6.7% [2 of 30], P = .23), although not statistically significant. Our previous RCT had proved the effectiveness of utilizing a brief intervention based on self-determination theory effective in promoting ED attendees quit smoking [25]. We conducted pilot study in a public hospital to prevent NCDs by screening for and educating on health-risk behaviours among ED attendees (N = 150). Although not significantly different, a higher proportion of participants in the intervention group reported a behavioural change than did those in the control group (38% vs. 26%) at 6-month follow-up. The results demonstrated the feasibility and potential of a general health-promotion intervention based on self-determination theory in helping ED attendees adopt healthy lifestyles. A large-scale RCT is warranted to obtain strong evidence of the efficacy of this approach.

Therefore, we propose the current study to examine the effectiveness of a general health promotion intervention based on self-determination theory in helping ED attendees adopt a healthy lifestyle. We hypothesize that a higher proportion of individuals in the intervention group will adopt healthy lifestyles and have a better health-related quality of life than those in the control group at 6- and 12-month follow-ups. The proposed intervention, if proved effective, will add to the existing evidence on promoting healthy lifestyles in busy clinical EDs, inform researchers and policymakers concerning the prevention and control of NCDs, and provide implications for clinical practice.

## Materials and methods

### Study design

**Fig 1:**
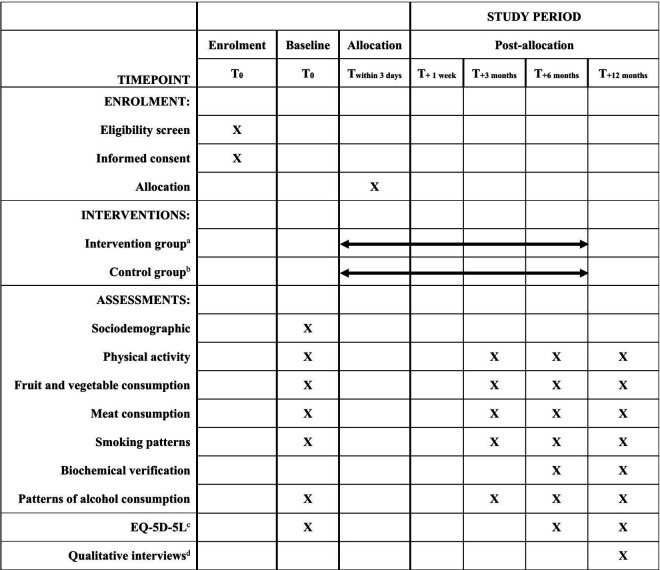
The SPIRIT schedule of enrolment, interventions, and assessments. ^a^ A brief telephone intervention using the AWARD model (Ask, Warn, Advise, Refer, Do-it-again), personalized health advice, weekly WhatsApp/WeChat reminders, and 1-minute educational videos, with follow-up assessments at 3, 6, and 12 months to encourage sequential or simultaneous lifestyle changes. ^b^ simplified AWARD-based telephone intervention with general health advice, regular SMS messages containing generic health tips, and follow-up assessments at the same intervals, but without personalized videos or interactive messaging support. ^c^ The EuroQoL 5-Dimension 5-level Questionnaire. ^d^ 20 purposely selected participants in the intervention group will be interviewed.

A single-blinded, multicentre RCT with a two-group between-subjects design will be conducted on 1,172 ED attendees at the EDs of five major acute care hospitals in different clusters in Hong Kong: (a) Queen Mary Hospital; (b) Prince of Wales Hospital; (c) Pamela Youde Nethersole Easten Hospital; (d) Kwong Wah Hospital; and (e) United Christian Hospital. A Standard Protocol Items: Recommendations for Interventional Trials (SPIRIT) [26] schedule of enrollment, interventions, and assessments is shown in Fig 1, and a completed SPIRIT checklist is shown in S1 File. The original study protocol submitted for IRB approval is shown in S2 File.

### Participants

Chinese adults who attend one of the five EDs in various clusters in Hong Kong because of physical discomfort and if they meet the following criteria will be invited to participate. The inclusion criteria will be as follows: (1) being aged ≥18 years, (2) being triaged as semi-urgent (level 4) or non-urgent (level 5) and discharged home on the same day after receiving medical attention, (3) having at least one health risk behaviour (tobacco use, harmful alcohol consumption, unhealthy diet, and physical inactivity), and (4) owning a smartphone and having an ability to use instant messaging applications (e.g., WhatsApp or WeChat). The specific criteria for each health risk behaviour are listed in Table 1.

**Table 1:** Criterion for identification of each health-risk behaviour and successful adopting a healthy lifestyle.

Behavioural risk factors	Criterion of health-risk behaviour	Successful criteria of adopting a healthy lifestyle
Tobacco use [27]	Smoked at least 1 cigarette a day over the past 30 days	Self-reported 7-day point prevalence of smoking abstinence
Binge drinking [22]	Reporting ≥1 binge drinking episodes (consumption of five or more alcoholic beverages on one occasion) in the past month.	Self-report no binge drinking episode (consumption of five or more alcoholic beverages on one occasion) in the past month.
Unhealthy diet [28]	Have consumed less than five servings of fruit and vegetables per day or have had a daily intake of less than 400 grams of fruit and vegetables	Have consumed at least five servings of fruit and vegetables per day or have had a daily intake of at least 400 grams of fruit and vegetables
Physical inactivity [29]	(1) Performed less than 150 minutes of moderate-intensity aerobic physical activity throughout the week, or (2) performed less than 75 minutes of vigorous-intensity aerobic physical activity throughout the week	Performed at least 150 minutes of moderate-intensity aerobic physical activity throughout the week, or performed at least 75 minutes of vigorous-intensity aerobic physical activity throughout the week

The Exclusion criteria will be as follows: (1) having a poor cognitive state or mental illness and (2) being diagnosed with NCDs and undergoing regular follow-ups in outpatient clinics, and (3) participating in another related study. People who do not have a smartphone or use WhatsApp/WeChat will be provided brief advice and a Practical Resource Hub for Healthy Life leaflet (S3 file) on healthy lifestyles but will be excluded from the study.

### Sample size

We used G*Power to estimate the sample size based on the results of our study conducted at an ED based on self-determination theory [25]. The results of the pilot study showed a greater event rate of adopting at least one healthy lifestyle at 6 months (the primary outcome of this proposed project) in the intervention group than the control group (38% vs 26%). By allowing for a more conservative effect (with between group difference of 35% vs 26% at 6 months), it is estimated that a sample size of 410 participants in each of the two groups are required to achieve 80% power at a 5% level of significance (two-tailed). To account for a potential attrition rate of 30% at the 6-month follow-up, we will recruit a total of 1,172 participants (586 per group).

### Subject recruitment

All potential participants will be approached by emergency nurses before being discharged from the EDs. These nurses will provide potential participants with a leaflet detailing the nature, purpose, design, procedures, and potential benefits and risks of the study. Subsequently, the emergency nurses will refer the potential participants to the RA. Informed written consent will be obtained from all participants. Participants will be assured that their participation will be voluntary, with no prejudice attached to refusal, and that the information provided by them will be kept confidential. A baseline assessment will be performed using questionnaires. Then, participants will be informed that they will receive a telephone call from an RA within 3 days to evaluate their potential health-risk behaviours and provide them with appropriate health advice to adopt healthy lifestyles. In addition, the participants will be provided a Practical Resource Hub for Healthy Life leaflet containing information on various applications, including (i) ‘Move Your Body’, (ii) ‘Eat Healthy’, (iii) ‘Live Alcohol Free’, and (iv) ‘Stay Away from Tobacco’, which were developed by the Hong Kong Department of Health (S3 File).

### Randomization

To prevent the potential risk of treatment contamination within the EDs, randomization will not be performed on site. Instead, the research assistant (RA) will input baseline data collected at the EDs directly into the web-based trial entry form linked to a computerised database. Subsequently, randomisation will be performed at the principal investigator’s institution by an independent statistician who will have no other involvement in the study. Stratified block randomization with 1:1 allocation will be conducted using varying block sizes of 4–10 to achieve an appropriate balance of participant numbers between the intervention and control groups to optimize allocation concealment.

### Intervention

The proposed intervention is guided by the theory of planned behaviour, the foot-in-the-door technique, and self-determination theory (S4 File). Our intervention aims to first change the participants’ attitudes and their subjective norms through risk communication. Using foot-in-the-door technique and self-determination theory, it will then increase participants’ willingness to adopt a healthy lifestyle.

### Intervention group

(a) Brief intervention via telephone (within 3 days after visiting the ED)

The participants will receive a brief intervention using the Ask, Warn, Advise, Refer and Do-it- again (AWARD) model, which was originally developed for primary-care tobacco cessation. This intervention includes the following steps: (1) Ask about and assess health-risk behaviours; (2) Warn about the high morbidity and mortality risks associated with health-risk behaviours; (3) Advise on adopting healthy lifestyles to improve the participant’s health; (4) Refer to hotline services, such as those for smoking cessation and alcohol treatment or the nearest district health centre to follow up their health status; and (5) Do it again if participants have not adopted a healthy lifestyle at follow-ups. For the advice step, the RA will ask about the participants’ priority in engaging in a desired health-related lifestyle based on their responses in the behavioural risk factor questionnaire. The participants will also be asked to choose a goal that they consider most attainable, such as quitting or reducing smoking, consuming more vegetables or less fatty foods or sugary drinks, performing more exercise, or reducing alcohol consumption. Although the participants will be encouraged to adopt aspects of a healthy lifestyle sequentially, they will have the option to adopt them simultaneously if they feel confident in doing so. Each participant will receive a brief (approximately 5 minutes) individual intervention providing health advice on their selected lifestyle goal. The entire intervention will last approximately 10 minutes and may be extended if necessary. At the end of the telephone call, the participant will be informed that the RA will assist them in achieving their health-related goals throughout the study by sending messages via WhatsApp/WeChat.

(b) Follow-up booster intervention (up to 6 months)

During the first 6 months of the proposed project, the RA will send WhatsApp/WeChat messages approximately once a week to remind the participants to adhere to their desired health-related lifestyle. Instant messaging via mobile applications was found to be effective in enhancing treatment compliance. In addition, during the first week, the RA will send participants a link via WhatsApp/WeChat to a 1-minute video developed by the research team comprising content relevant to their selected health-related lifestyle. Four separate 1-minute videos will be compiled, each focusing on a different healthy lifestyle. These videos will indicate the health hazards of continuing this health risk behaviour and the benefits of adopting a healthy lifestyle. Moreover, the RA will encourage the participants to watch the video and ask any questions regarding the video content via WhatsApp/WeChat. One advantage of using videos to deliver instant health advice messages is the use of sound and images, which can elicit emotions, enhance understanding of abstract concepts, and improve the retention of new information through auditory, visual, and verbal stimulation. Moreover, the delivered content can be viewed by the participants at their convenience and own pace.

(c) Follow-up assessments at 3, 6, and 12 months The success of the participants in achieving their targeted health-related lifestyle will be assessed through phone calls at 3, 6, and 12 months. If the participants report the successful adoption of a healthy lifestyle, the RA will encourage them to adopt another healthy lifestyle. Then, the RA will provide participants with brief healthcare advice (approximately 5 minutes) and send via WhatsApp/WeChat another 1-minute video focused on their newly chosen and desired health-related lifestyle.

### Control group

Participants will receive a brief telephone intervention based on the AWARD model from the trained RA, similar to that delivered to the intervention group. However, the RA will only advise the participants to adopt a healthy lifestyle. In addition, the RA will send regular SMS messages to participants at a frequency similar to that used for the intervention group. However, these messages will contain only general health advice. In addition, the participants will receive follow-up outcome assessments at the same schedule as that followed in the intervention group.

### Outcome assessments and measurements

The primary outcome will be the composite event rate of adopting at least one of the four healthy lifestyles specified in Table 1 assessed at 6 months. The secondary outcomes will be the composite event rate of adopting at least one of the four healthy lifestyles assessed at 12 months and improvement in health-related quality of life assessed at 6 and 12 months.

A behavioural risk-factor questionnaire will be used as the patient-report outcome measure to collect data on eligible participants’ demographic and health-risk behaviours (tobacco use, binge drinking, unhealthy diet and physical inactivity) at baseline and at 3, 6, and 12 months (S5 File). Moreover, participants’ blood pressure and body mass index will be documented.

The EuroQoL 5-Dimension 5-level (EQ-5D-5L) [30] will be used to assess the participants’ health-related quality of life at baseline, 6 months, and 12 months (S6 File). The psychometric properties of the Chinese version of the EQ-5D-5L were tested, and the findings indicate that this tool is a valid, reliable, and sensitive measure of health-related quality of life [31]. A Chinese-specific EQ-5D-5L value set will enable the calculation of health utility scores applicable to the Chinese population and quality-adjusted life-years (QALYs) for conducting cost-effectiveness analysis [31].

We will use available objective assessment tools to supplement the participants’ self-reported outcomes to determine changes in their health-risk behaviours after the intervention. For verifying smoking cessation, we will use the biochemically validated 7-day point prevalence of abstinence, determined by a saliva cotinine level of <30 ng/ml and an exhaled carbon monoxide level below <4 ppm. Only those who meet both these criteria will be regarded as biochemically validated abstinent individuals; otherwise, the participants will be considered to have failed the validation. In addition, those participants who stop smoking but continue to use medicinal nicotine or e-cigarettes are considered unsuccessful quitters. For participants on nicotine replacement therapy, biochemical validation will be conducted 7 days after the completion of therapy.

### Quality and data security control

The PI, Co-Is, project coordinator, and RAs will organise meetings with the chief of service, nurse managers, and frontier nurses to explain the intervention protocol and study logistics and examine the physical facilities available at the EDs. All blank and completed questionnaires will be sealed in separate opaque envelopes and stored in a locker with keys provided by the EDs. The project coordinator will manually collect the completed questionnaires on a weekly basis, and they will be stored in a locker (with the keys kept in our department). Only the PI, Co-Is, project coordinator, relevant RAs, the independent data monitoring committee, and the institutional review boards will have access to the collected data.

### Statistical analysis

Both assessors and the analyst will be blinded to the assigned treatment group of the participants. The baseline characteristics of the two groups will first be compared using the chi-square test for categorical variables and analysis of variance (ANOVA) for continuous variables. An intention-to-treat analysis will be used by imputing all non-responses at follow-up by baseline values (i.e., assuming failure or no change after the intervention), to yield more conservative effect size estimates. SPSS for Windows (SPSS version 26.0; IBM Corp., Armonk, NY, USA) will be used for the quantitative data analysis with level of significance set at 0.05 (two-tailed).

Descriptive statistics, such as mean, standard deviation, frequency and percentage will be used to present the baseline demographic and clinical characteristics, and outcome variables, including health-risk behaviour data, across the study time points. As our study is a RCT which is expected to control all observed and even unknown confounding factors, the primary analysis for the outcomes will therefore be based on unadjusted analysis. A logistic regression analysis will be used to compare the composite event rate of adopting at least one of the four healthy lifestyles at 6 months (primary outcome) and at 12 months (secondary outcome) between the intervention and control groups. A generalised estimating equations (GEE) model will be used to compare the differential changes in health-related quality of life at 6 and 12 months with respect to baseline (secondary outcome) between groups. Sensitivity analyses will be conducted (1) with adjustment for the baseline demographic and clinical characteristics that show significant differences between groups, and (2) by using multiple imputation via a fully conditional specification method to handle missing data to evaluate the robustness of the primary analysis results. The Holm’s multiplicity adjustment procedure will be used to control family-wise type I error rate for assessing the significance of each outcome repeatedly assessed at 6 and 12 months. The effectiveness of the intervention will be established if the intervention group shows significantly higher event rate of adopting at least one of the four healthy lifestyles at 6 months (primary outcome) than the control group. Cost-effectiveness analysis (CEA) will be conducted (S7 File).

We will use a qualitative approach to examine participants’ experiences after receiving the intervention. Based on the number of healthy lifestyles they adopt at 12 months, 20 participants from the intervention group (10 with a higher number of healthy lifestyles they adopt and 10 with a lower number) will be interviewed. The final sample size will depend on the data saturation. An in-depth, one-on-one, audiotaped, semi-structured interview will be conducted with each participant. The data analysis process will begin immediately after each individual interview, in accordance with the thematic analysis framework introduced by Braun and Clarke [32], using NVivo v12 (2018; QSR International Pty Ltd, Melbourne, Australia). The codes, categories, and themes generated through this process will be compared.

### Ethics

All the procedures in this clinical trial will be conducted as per the ethical principles of the Declaration of Helsinki. Ethical approval was obtained from the Chinese University of Hong Kong-New Territories East Cluster (CUHK-NTEC) Clinical Research Ethics Committee (CREC) on 27 November 2024, with a CREC Reference number of 2024.492-T. Written informed consent will be taken from each participant after their approval.

### Safety considerations

Participants will be carefully monitored for any potential complications due to lifestyle changes during each follow-up call. We will proactively refer participants with complications to medical physicians in EDs. They will then be considered by the research team whether they should be suitable to continue or withdraw from the study.

### Trial status

As of the time of this publication, the study is actively underway in accordance with protocol version 2, dated March 25, 2025. Recruitment of participants will be started on June 10, 2025, and is anticipated to be completed by December 30, 2026. The intervention phase and the last follow-up assessments for primary and secondary outcomes are expected to be completed by January 31, 2028. The study is progressing according to the planned timeline, and data analysis will be started after the completion of primary outcome measurements.

## Discussion

Sufficient medical evidence has shown that most premature deaths from NCD are preventable through lifestyle modification – quitting smoking, avoiding alcohol, having a balanced diet and engaging in regular physical activity [14]. There has been suggestion that eliminating health risk behaviours would prevent 80% of heart disease, stroke, type 2 diabetes and 40% of cancers [33,34]. Therefore, by helping people adopt healthy lifestyles, NCDs can be prevented, and overall health of the population can be improved. There is a lack of proactive approaches for screening and educating individuals with health-risk behaviours to prevent NCDs when they seek care, especially for the Chinese population. Presenting at the EDs and seeking medical treatment from doctors is a critical teachable moment to intervene. This study is the first clinical trial to examine the effectiveness of a proactive health promotion intervention based on self-determination theory through screening and educating ED attendees in helping them adopt a healthy lifestyle, as well as in improving the quality of life. The 6-month intervention is expected to gradually and positively influence the health behaviors of participants by helping them adopting the behaviours one by one through the establishment of self-confidence in the process of successfully adopting one of the health behavours. In addition, healthy lifestyles adoption is expected to positively influence their quality of life and mental health. Our study results will also demonstrate the cost-effectiveness of the intervention, potentially be widely accessible and affordable across the EDs in hospitals in Hong Kong. For the first time, this trial target on changing several lifestyle risk factors of NCDs using a proactive theory-based approach. The study will provide insights for the policymakers, clinical practitioners, and researchers into the proactive approaches that are effective in promoting healthy lifestyles and reducing risks of NCDs in the settings of EDs.

## Supporting information

S1 File SPIRIT 2013 Checklist: Recommended items to address in a clinical trial protocol and related documents. (DOCX)

S2 File Study Protocol. (PDF)

S3 File Practical Resource Hub for Healthy Life Leaflet. (PDF)

S4 File Theoretical framework. (DOCX)

S5 File Behaviour risk factor survey. (PDF)

S6 File EQ-5D-5L. (PDF)

S7 File Cost-effectiveness analysis (CEA). (DOCX)
