# Supplementary material for: A proactive approach to prevent non-communicable diseases through screening and educating emergency department attendees to adopt healthy lifestyles: Study protocol for a pragmatic, multicenter, randomized controlled trial
Source: PLoS One. 2025 Jul 3;20(7):e0327558. doi: 10.1371/journal.pone.0327558 (PMC12225783; doi:10.1371/journal.pone.0327558)
Supplement: S2 File — (PDF) [file pone.0327558.s002.pdf]

Title:
A proactive approach to prevent non-communicable diseases through screening and educating emergency department attendees to adopt healthy lifestyles: A randomised clinical trial

Introduction:

Hong Kong is facing a growing burden of non-communicable diseases (NCDs), such as cardiovascular diseases, cancer, diabetes, and chronic respiratory diseases, exacerbated by an aging population.[1,2] The World Health Organization has identified four major behavioural risk factors that substantially contribute to NCDs and can increase the risk of death: tobacco use, excessive alcohol consumption, an unhealthy diet, and physical inactivity.[3] Most premature deaths from NCDs could have been prevented through lifestyle modifications.[3,4]

In 2021, we conducted a large population survey (N = 5,737) in all 18 districts of Hong Kong to investigate the health-risk behaviours of Chinese adults.[5,6] The results showed that 80.3% of the participants had at least one health-risk behaviour, and 47.0% of the participants had two or more health-risk behaviours.[5,6] In response to the challenges posed by the increasing prevalence of NCDs, the government has committed to enhancing district-based primary healthcare services by establishing health centres in all districts across Hong Kong and launching the Chronic Disease Co-Care Pilot Scheme in 2023.[7] However, many Chinese adults lack motivation or experience difficulty in adopting a healthy lifestyle, especially if they do not receive advice or support from healthcare professionals.[8-11] In addition, the survey findings revealed that more than half of the participants had not undergone regular body check-ups or monitored their physical health at home in the past 3 years.[6] Although regular body check-ups are crucial for preventing NCDs,[12] a common misconception among many Chinese adults is that they do not need physical check-ups if they feel well.[13] Thus **a proactive approach is required** to screen people with health-risk behaviours and help them adopt healthy lifestyles, including quitting smoking, avoiding excessive alcohol consumption, maintaining a balanced diet, and engaging in regular physical activity.

The receipt of medical attention at an ED by a person in physical discomfort can serve as an excellent 'teachable moment' because it provides an invaluable opportunity to initiate a healthy lifestyle. People who consult a doctor in an ED are more likely to modify their risk behaviours to improve their health. According to the Hospital Authority Annual Report,[14] there were 1.8 million ED visits in Hong Kong between 2021 and 2022. More than half of all ED visits were triaged as semi-urgent (level 4) or non-urgent (level 5), and most of the patients were discharged home after receiving medical attention. A previous study found that 86.6% of patients admitted to and discharged from the ED on the same day had a high risk of NCDs, such as blood pressure exceeding 160/100 mmHg before discharge.[15] Furthermore, more than 75% of these patients did not receive any referral or follow-up care.[15] The proposed project will address the gaps in the literature and will align with the Health Bureau's thematic priority B-0002: 'Develop and test novel approaches for enhancing prevention, early diagnosis, treatment, and recovery/ rehabilitation from major NCD'.

Aim and Hypotheses to be Tested:

Aim
To examine the effectiveness of a general health promotion intervention based on self-determination theory in helping ED attendees adopt a healthy lifestyle.

Hypothesis to be tested

We hypothesise that a higher proportion of individuals in the intervention group will adopt healthy lifestyles and have a better health-related quality of life than those in the control group at 6- and 12-month follow-ups.

Plan of Investigation:

Study design

An assessor-blinded, multicentre RCT with a two-group between-subjects design will be used following the Consolidated Standards of Reporting Trial.

Randomisation and allocation concealment

To prevent the potential risk of treatment contamination within the ED, randomisation will not be performed there. Instead, the research assistant (RA) will input baseline data collected at the ED directly into the web-based trial entry form linked to a computerised database. Subsequently, randomisation will be performed at the principal investigator's institution by an independent statistician who will have no other involvement in the study. Stratified block randomisation with 1:1 allocation will be conducted using varying block sizes of 4–10 to achieve an appropriate balance of participant numbers between the intervention and control groups and to optimise allocation concealment.

Methods

Setting:

The proposed project will be conducted at the EDs of five major acute care hospitals in different clusters in Hong Kong. The source of funding for this project is Health and Medical Research Fund (HMRF), Health Bureau.

Conceptual and theoretical framework:

The proposed intervention is guided by the theory of planned behaviour, the foot-in-the-door technique, and self-determination theory.

Theory of planned behaviour

This theory[16] holds that an intention to engage in a health-related behaviour is determined by the proximal factors of attitudes, subjective norms, and perceived behavioural control. Attitudes represent individuals perceived likelihood of performing and appraisal of the consequences of performing a health-promoting behaviour. Subjective norms are the social pressure perceptions to either perform or not perform a health-promoting behaviour. Finally, perceived behavioural control is an individual's perception of control regarding performing the behaviour.

Foot-in-the-door technique

The foot-in-the-door technique, which was introduced by Freeman and Fraser,[17] emphasises the notion that individuals who initially comply with a small, easy request are more likely to later comply with a larger request.[17] Compliance with the first request or target increases the individual's confidence and alters their self-perceived capability and willingness regarding further requests or targets. This technique can facilitate the recruitment process and enhance compliance.

Self-determination theory

According to self-determination theory,[18] behavioural regulation is more autonomous when it is internalised, as opposed to being regulated by external factors. Compared with external regulation, autonomous regulation is associated with increased self-efficacy, greater behavioural persistence, longer-term

behavioural changes and more positive health behaviour.[24] Autonomy is another influential determinant of behaviour that is emphasised by freedom of choice.[18] There is some evidence that people who have greater autonomy demonstrating greater competence and self-efficacy in achieving behavioural change compared with those with less autonomy.[18-20] As a result, increased autonomy will facilitate a gradual change in risky behaviours.

Our intervention aims to first change the participants' attitudes and their subjective norms through risk communication. Using foot-in-the-door technique and self-determination theory, it will then increase participants' willingness to adopt a healthy lifestyle.

Recruitment

At the EDs

We will obtain ethical approval from the institutional review boards of the five hospitals. **All** potential participants will be approached by emergency nurses before being discharged from the EDs. These nurses will provide potential participants with a leaflet detailing the nature, purpose, design, procedures, and potential benefits and risks of the study. Subsequently, the emergency nurses will refer the potential participants to the RA. Informed written consent will be obtained from all participants. Participants will be assured that their participation will be voluntary, with no prejudice attached to refusal, and that the information provided by them will be kept confidential. A baseline assessment will be performed using questionnaires. Then, participants will be informed that they will receive a telephone call from an RA within 3 days to evaluate their potential health-risk behaviours and provide them with appropriate health advice to adopt healthy lifestyles. In addition, the participants will be provided a Practical Resource Hub for Healthy Life leaflet (Appendix 2) containing information on various applications, including (i) 'Move Your Body', (ii) 'Eat Healthy', (iii) 'Live Alcohol Free', and (iv) 'Stay Away from Tobacco', which were developed by the Hong Kong Department of Health.

Intervention

(a) Intervention group

Brief intervention via telephone (within 3 days after visiting the ED)

The participants will receive a brief intervention using the Ask, Warn, Advise, Refer and Do-it-again (AWARD) model, which was originally developed for primary-care tobacco cessation.[9,10,24] This intervention includes the following steps: (1) **A**sk about and assess health-risk behaviours; (2) **W**arn about the high morbidity and mortality risks associated with health-risk behaviours; (3) **A**dvise on adopting healthy lifestyles to improve the participant's health; (4) **R**efer to hotline services, such as those for smoking cessation and alcohol treatment or the nearest district health centre to follow up their health status; and (5) **D**o it again if participants have not adopted a healthy lifestyle at follow-ups. For the advice step, the RA will ask about the participants' priority in engaging in a desired health-related lifestyle based on their responses in the behavioural risk factor questionnaire. The participants will also be asked to choose a goal that they consider most attainable, such as quitting or reducing smoking, consuming more vegetables or less fatty foods or sugary drinks, performing more exercise, or reducing alcohol consumption. Although the participants will be encouraged to adopt aspects of a healthy lifestyle sequentially, they will have the option to adopt them simultaneously if they feel confident in doing so. Each participant will receive a brief (approximately 5 minutes) individual intervention providing health advice on their selected lifestyle goal. The entire intervention will last approximately 10 minutes and may be extended if necessary.

At the end of the telephone call, the participant will be informed that the RA will assist them in achieving their health-related goals throughout the study by sending messages via WhatsApp/WeChat.

Follow-up booster intervention (up to 6 months)

During the first 6 months of the proposed project, the RA will send WhatsApp/WeChat messages approximately once a week to remind the participants to adhere to their desired health-related lifestyle. Instant messaging via mobile applications was found to be effective in enhancing treatment compliance.[21]

In addition, during the first week, the RA will send participants a link via WhatsApp/WeChat to a 1-minute video developed by the research team comprising content relevant to their selected health-related lifestyle. Four separate 1-minute videos will be compiled, each focusing on a different healthy lifestyle. These videos will indicate the health hazards of continuing this health risk behaviour and the benefits of adopting a healthy lifestyle. Moreover, the RA will encourage the participants to watch the video and ask any questions regarding the video content via WhatsApp/WeChat. One advantage of using videos to deliver instant health advice messages is the use of sound and images, which can elicit emotions, enhance understanding of abstract concepts, and improve the retention of new information through auditory, visual, and verbal stimulation.[22-24] Moreover, the delivered content can be viewed by the participants at their convenience and own pace.

Follow-up assessment of behavioural changes at 3, 6, and 12 months

The success of the participants in achieving their targeted health-related lifestyle will be assessed through phone calls at 3, 6, and 12 months. If the participants report the successful adoption of a healthy lifestyle, the RA will encourage them to adopt another healthy lifestyle. Then, the RA will provide participants with brief healthcare advice (approximately 5 minutes) and send via WhatsApp/WeChat another 1-minute video focused on their newly chosen and desired health-related lifestyle.

(b) Control group

Participants will receive a brief telephone intervention based on the AWARD model from the trained RA, similar to that delivered to the intervention group. However, the RA will only advise the participants to adopt a healthy lifestyle. In addition, the RA will send regular SMS messages to participants at a frequency similar to that used for the intervention group. However, these messages will contain only general health advice. In addition, the participants will receive follow-up outcome assessments at the same schedule as that followed in the intervention group.

Training and quality assurance

Prior to the study initiation, the PI and Co-Investigators (Co-Is) will conduct a training workshop for the RAs. The training will equip the RAs with the necessary knowledge and skills required to deliver a brief intervention based on the AWARD model and provide healthcare advice on various health-related lifestyles. Regular case conferences and quality assessments will be conducted through audiotaping, and audits will be conducted to ensure the quality and uniformity of the interventions.

Subjects

Chinese adults who attend one of the five EDs in various clusters in Hong Kong because of physical discomfort and if they meet the following criteria will be invited to participate. The inclusion criteria will be as follows: (1) being aged ≥18 years, (2) being triaged as semi-urgent (level 4) or non-urgent (level 5) and discharged home on the same day after receiving medical attention, (3) having at least one health risk behaviour (tobacco use, harmful alcohol consumption, unhealthy diet, and physical inactivity), and (4) owning a smartphone and having an ability to use instant messaging applications (e.g. WhatsApp or WeChat). The Exclusion criteria will be as follows: (1) having a poor cognitive state or mental illness and (2) being diagnosed with NCDs and undergoing regular follow-ups in outpatient clinics, and (3) participating in another related study.

The specific criteria for each health risk behaviour are listed in Appendix 1. People who do not have a smartphone or use WhatsApp/WeChat (unlikely in Hong Kong) will be provided brief advice and a Practical Resource Hub for Healthy Life leaflet on healthy lifestyles but will be excluded from the study.

Sample size

We used G*Power to estimate the sample size based on the results of our pilot study conducted at an ED and a previous study using a brief smoking cessation intervention based on self-determination theory.[24] The results of the pilot study showed an approximately 12% between-group difference in successfully adopting at least one healthy lifestyle at 6 months (the primary outcome of this proposed project). However, we opted for a more conservative effect size for our sample size calculation. Based on our previous large-scale RCT on a brief smoking cessation intervention based on self-determination theory,[24] we expect a between-group difference of at least 5% (≥10% in the intervention group vs. ≤5% in the control group) in the primary outcome at 6 months. To detect a significant between-group difference with a power of 80% at a significance level of 5% (two-tailed) in the proportion of participants modifying their health risk behaviours and/or adopting healthy lifestyles, 435 participants will be needed per group. To account for a potential attrition rate of 30% at the 6-month follow-up, we will recruit a total of 1,242 participants.

Based on the number of EDs visits (1.8 million) in Hong Kong between 2021 and 2022,[25] we are confident that we will be able to recruit the required number of participants within 18 months.

Data processing and analysis

Instruments:

We will use available objective assessment tools to supplement the participants' self-reported outcomes to determine changes in their health-risk behaviours after the intervention. For verifying smoking cessation, we will use the biochemically validated 7-day point prevalence of abstinence, determined by a saliva cotinine level of <115 ng/ml and an exhaled carbon monoxide level below <4 ppm. Only those who meet both these criteria will be regarded as biochemically validated abstinent individuals; otherwise, the participants will be considered to have failed the validation. HK$200 supermarket coupon being the incentive for completing biochemical validations

A behavioural risk-factor questionnaire will be used as the patient-report outcome measure to collect data on eligible participants' demographic and health-risk behaviours (tobacco use, binge drinking, unhealthy diet and physical inactivity) at baseline and at 3, 6, and 12 months. Moreover, participants' blood pressure and body mass index will be documented. This questionnaire was used to investigate multiple health-risk behaviours among Chinese adults in Hong Kong in 2021.[5]

The EuroQoL 5-Dimension 5-level (EQ-5D-5L)[25] will be used to assess the participants' health-related quality of life at baseline, 6 months, and 12 months. The psychometric properties of the Chinese version of the EQ-5D-5L were tested, and the findings indicate that this tool is a valid, reliable, and sensitive measure of health-related quality of life.[26] A Chinese-specific EQ-5D-5L value set will enable the calculation of health utility scores applicable to the Chinese population and quality-adjusted life-years (QALYs) for conducting cost-effectiveness analysis.[26]

Outcome measures:

The **primary outcome** will be the number of healthy lifestyles adopted at 6 months. The criteria of successful adopting a healthy lifestyle are presented in Appendix 1.

The secondary outcomes will be the number of healthy lifestyles adopted at 12 months and improvement in health-related quality of life at 6 and 12 months.

Data Analysis

The baseline characteristics of the two groups will first be compared using the chi-square test for categorical variables and analysis of variance for continuous variables. An intention-to-treat analysis will be used by imputing all non-responses at follow-up by baseline values (i.e. assuming failure or no change after the intervention), to yield more conservative effect size estimates.

SPSS for Windows (SPSS version 26.0; IBM Corp., Armonk, NY, USA) will be used for the quantitative data analysis. Descriptive statistics will be used to calculate the mean, standard deviation, and frequency of the demographic and health-risk behaviour data. The primary analysis including the main effect, i.e., behavioural changes at 6 months in the intervention group versus those in the control group, will be performed using a chi-square test or Fisher's exact test. The secondary analyses will (1) assess the main effect adjusted for baseline differences, (2) assess the secondary outcomes (health-related quality of life) at 6 and 12 months, and (3) construct a generalised estimating equation model (GEE). The GEE model will be used to calculate the adjusted odds ratios for the self-reported the number of healthy lifestyles engaged in, after adjusting for the baseline demographic and clinical characteristics that show significant differences, and the within-subjects effect of the repeated-measure outcomes (6 and 12 months).

We will conduct a cost-effectiveness analysis (CEA) using standard methods.[27] The CEA will be populated using empirical 12-month RCT data. An ingredients approach will be used to estimate the cost of the intervention programme, including that of the intervention material (e.g., leaflets and videos), the administration cost, and the time taken to deliver the intervention. The health effectiveness outcomes will include the number of participants who have adopted healthy lifestyles at 6 and 12 months and their QALYs.

We will use a qualitative approach to examine participants' experiences after receiving the general health promotion intervention based on self-determination theory. Based on the number of healthy lifestyles they adopt at 12 months, 20 participants from the intervention group (10 with a higher number of healthy lifestyles they adopt and 10 with a lower number) will be interviewed. The final sample size will depend on the data saturation. An in-depth, one-on-one, audiotaped, semi-structured interview will be conducted with each participant. The data analysis process will begin immediately after each individual interview, in accordance with the thematic analysis framework introduced by Braun and Clarke,[28] using NVivo v12 (2018; QSR International Pty Ltd, Melbourne, Australia). The codes, categories, and themes generated through this process will be compared. HK$200 supermarket coupon as an incentive to participants who have completed in-depth interview.

Ethical Concern

This research project is in compliance with the Declaration of Helsinki.

To protect participants' privacy, all research data would be handled in line with HA / Hospital's policy in handling / storage / destruction of patients' medical records. They would be locked in cabinets to keeps patients' information in confidential. Electronic data should be saved in secured computer with restricted access. The protocol complies with the Declaration of Helsinki.. All research and personal data will be kept for 5 years upon study completion. All personal and study data will be used for the purpose of this study. It will be discarded in accordance to the prevailing data management policy after the aforesaid storage period.

Key References:

1. Hong Kong. Food Health Bureau, issuing body. *Towards 2025: Strategy and Action Plan to Prevent and Control Non-Communicable Diseases in Hong Kong*. [Department of Health], 2018.
2. 2016 Statistics of Inpatient Discharges and Deaths. Hong Kong SAR: Hospital Authority, Department of Health and Census and Statistics Department.
3. World Health Organization, Global Action Plan for the Prevention and Control of Noncommunicable Diseases 2013-2020. 2013. Available online: http://apps.who.int/iris/bitstream/10665/94384/1/9789241506236_eng.pdf?ua=1. (accessed on 1 May 2023)
4. GBD 2015 Risk Factors Collaborators. Global, regional, and national comparative risk assessment of 79 behavioural, environmental and occupational, and metabolic risks or clusters of risks, 1990–2015: a systematic analysis for the Global Burden of Disease Study 2015. Lancet, 2016; 388(10053):1659-1724.
5. Li HCW, Ho LLK, Chung OKJ, Cheung AT, Xia W, Song P. A. Descriptive Study on Multiple Health-Risk Behaviors among Chinese Adults in Hong Kong. Int J Environ Res Public Health. 2022; 19(18): 11393.
6. Li HCW, Ho LLK, Cheung AT, Xia W, Song P. A, Chung OKJ. Health-risk behaviors among Chinese adults during COVID-19 pandemic. Int J Environ Res Public Health. 2023; 20: 2157.
7. Health Bureau. Available online: https://www.primaryhealthcare.gov.hk/cdcc/en/ (accessed on 1 February 2024).
8. Li HCW, Ho KY, Xia VW, Wang MP, Lam KK, Chan SS, Lam TH. Helping hospitalized smokers in Hong Kong quit smoking by understanding their risk perception, behaviour, and attitudes related to smoking. J Adv Nurs. 2019;75(10):2167-77.
9. Li HCW, Wang MP, Ho KY, Lam KWK, Cheung YTD, Cheung TY, Lam TH, Chan SSC. Helping cancer patients quit smoking using brief advice based on risk communication: A randomized controlled trial. Sci Rep. 2018, 8:2712.
10. Li HCW, Wang MP, LAM TH, Cheung TY, Cheung YTD, Suen YN, Ho KY, Tan KCB, Chan SSC. Brief intervention to promote smoking cessation and improve glycemic control in smokers with type 2 diabetes: a randomized controlled trial. Sci Rep. 2017; 7: 45902.
11. Chan SS, Leung DY, Wong DC, Lau CP, Wong VT, Lam TH. A randomized controlled trial of stage-matched intervention for smoking cessation in cardiac out-patients. Addiction.
12. Centers for Disease Control and Prevention (CDC). How You Can Prevent Chronic Diseases. 2021. Available online: https://www.cdc.gov/chronicdisease/about/index.htm. (accessed on 12 January 2024)
13. Wang, D.; Dai, X.; Mishra, S.R.; Lim, C.C.; Carrillo-Larco, R.M.; Gakidou, E.; Xu, X. Association between socioeconomic status and health behaviour change before and after non-communicable disease diagnoses: A multicohort study. Lancet Public Health 2022, 7, e670–e682.
14. Hospital Authority. "Hospital Authority annual report 2021–2022." (2022).
15. Graham CA, Chan SS, Ahmad I, Rainer TH. 405 Elevated Blood Pressure in Emergency Department Patients in Hong Kong: Prevalence, Characteristics and Disposition. Annals of Emergency Medicine, 2012; 60(4): S142-S143.
16. Ajzen I. The theory of planned behavior. Organ Behav Hum. 1991; 50(2): 179-211.
17. Freedman JL, Fraser SC. Compliance without pressure: the foot-in-the-door technique. J Pers Soc Psychol.1966; 4:195.
18. Deci E, Ryan R. *Handbook of self-determination research*. Rochester, NY: University of Rochester Press, 2002.
19. Bandura A. *Self-efficacy: The exercise of control*. New York: Freeman, 1997.
20. Williams GC, McGregor HA, Zeldman A, *et al*. Testing a self-determination theory process model for promoting glycemic control through diabetes self-management. *Health Psychology* 2004; **23**: 58-66.
21. Krishna R. The Impact of Health Information Technology on the Doctor-Patient Relationship in Child and Adolescent Psychiatry. Child Adol Psych Cl. 2017; 26: 67-75.
22. Xia W, Li H C W, Cai W, et al. Effectiveness of a video-based smoking cessation intervention focusing on maternal and child health in promoting quitting among expectant fathers in China: A randomized controlled trial. PLos Med. 2020; 17(9): e1003355.
23. Tuong W, Larsen ER, Armstrong AW. Videos to influence: a systematic review of effectiveness of video-based education in modifying health behaviors. J Behav Med. 2014; 37:218–233.
24. Dror I, Schmidt P, O'Connor L. A cognitive perspective on technology enhanced learning in medical training: great opportunities, pitfalls and challenges. Med Teach. 2011; 33:291–296.

25. Luo N, Liu G, Li M, Guan H, Jin X, Rand-Hendriksen K. Estimating an EQ-5D-5L Value Set for China. Value Health. 2017; 20(4):662-9.
26. Wong E L Y, Ramos-Goni J M, Cheung A W L, et al. Assessing the use of a feedback module to model EQ-5D-5L health states values in Hong Kong. The Patient-Patient-Centered Outcomes Research, 2018, 11(2): 235-247.
27. Cromwell J, Bartosch WJ, Fiore MC, Hasselblad V, Baker T. Cost-effectiveness of the clinical practice recommendations in the AHCPR guideline for smoking cessation. JAMA. 1997; 278:1759-66.
28. Braun V, Clarke V. Using thematic analysis in psychology. Qual Res Psychol. 2006; 3(2):77-101.

Appendix 1

Criterion for determination of each health-risk behaviour and criteria of successful adopting a healthy lifestyle

**Behavioural risk factors**	**Criterion of health-risk behaviour**	**Successful criteria of adopting a healthy lifestyle**
Tobacco use[1]	Smoked at least 1 cigarette a day over the past 30 days	Self-reported 7-day point prevalence of smoking abstinence
Binge drinking[2]	Reporting ≥1 binge drinking episodes (consumption of five or more alcoholic beverages on one occasion) in the past month.	Self-report no binge drinking episode (consumption of five or more alcoholic beverages on one occasion) in the past month.
Unhealthy diet[3]	Have consumed less than five servings of fruit and vegetables per day or have had a daily intake of less than 400 grams of fruit and vegetables	Have consumed at least five servings of fruit and vegetables per day or have had a daily intake of at least 400 grams of fruit and vegetables
Physical inactivity[4]	(1) Performed less than 150 minutes of moderate-intensity aerobic physical activity throughout the week, or (2) performed less than 75 minutes of vigorous-intensity aerobic physical activity throughout the week	Performed at least 150 minutes of moderate-intensity aerobic physical activity throughout the week, or performed at least 75 minutes of vigorous-intensity aerobic physical activity throughout the week

References

1. Wang MP, Suen YN, Li WH, et al. Intervention with brief cessation advice plus active referral for proactively recruited community smokers: a pragmatic cluster randomized clinical trial. JAMA Intern Med. 2017;177(12):1790-1797.
2. Lam TH, Chan B, Ho SY, Chan WM. Stage of change for general health promotion action and health-related lifestyle practices in Chinese adults. Preventive Medicine. 2004; 1;38(3):302-8.
3. Fruit, vegetables and NCD disease prevention. Geneva: World Health Organization; 2003. (https://www.who.int/news-room/fact-sheets/detail/healthy-diet).
4. Global Recommendations on Physical Activity for Health", World Health Organization; 2010. (http://whqlibdoc.who.int/publications/2010/9789241599979_eng.pdf)
