# Supplementary material for: A proactive approach to prevent non-communicable diseases through screening and educating emergency department attendees to adopt healthy lifestyles: Study protocol for a pragmatic, multicenter, randomized controlled trial
Source: PLoS One. 2025 Jul 3;20(7):e0327558. doi: 10.1371/journal.pone.0327558 (PMC12225783; doi:10.1371/journal.pone.0327558)
Supplement: S3 File — (PDF) [file pone.0327558.s003.pdf]

身心健康實用資訊站
Practical Resource Hub for Healthy Life

衛生署
Department of Health

邁向2025：香港非傳染病防控策略及行動計劃
Towards 2025: Strategy and Action Plan to Prevent and Control Non-communicable Diseases in Hong Kong

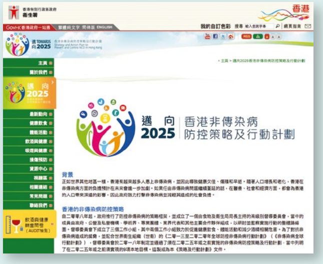

中文

English

心情鬆一鬆
Relax Your Mind

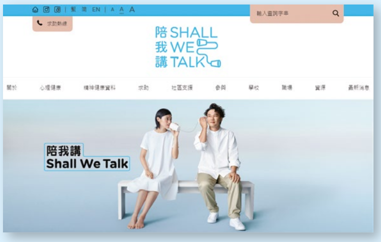

「陪我講」網站
“Shall We Talk” Website

中文

English

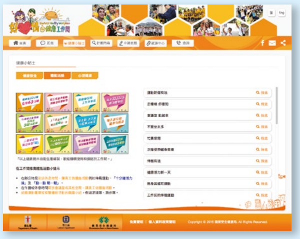

好心情@健康工作間網站
Joyful@Healthy Workplace Website

中文

English

每日動一動
Move Your Body

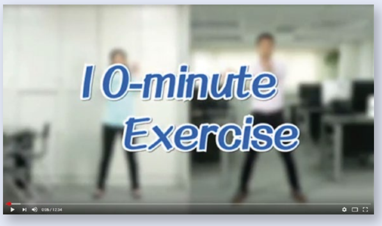

「十分鐘 活力操」
10-minute Exercise

中文字幕

English subtitle

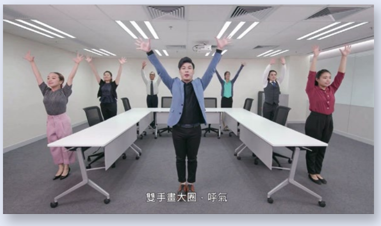

「動一動 鬆一鬆」
Let’s Take an Energetic Break

中文字幕

English subtitle

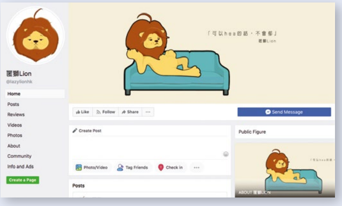

懶獅Lion
Facebook專頁
Lazylion Facebook Page

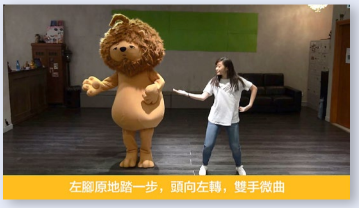

《郁一郁》全首舞蹈教學短片
“Move For Health”
Dance Tutorial (Full version)

中文字幕

English subtitle

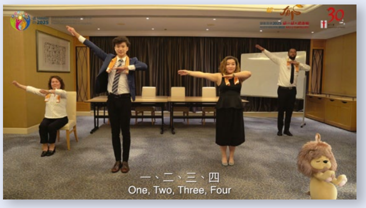

衛生署《郁一郁》三段操
“Move For Health” 3-Level Dances

簡易篇-日常伸展操
Lv1-Stretching Dance

進階篇-家居健體操
Lv2-Household Fitness Dance

挑戰篇-活力操
Lv3-Lively Dance

有「營」飲食
Eat Healthily

iOS

Android

「星級有營食肆」
流動應用程式
EatSmart
Restaurant Star+
Mobile Application

iOS

Android

營廚雜誌：
有「營」食譜
流動應用程式
CookSmart:
EatSmart Recipes
Mobile Application

iOS

Android

「小食紅黃綠」
流動應用程式
“Snack Check”
Mobile Application

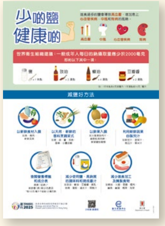

少啲鹽健康啲
Less Salt for
Better Health

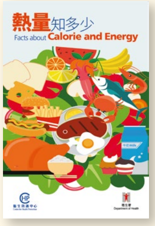

熱量知多少
Facts about Calorie
and Energy

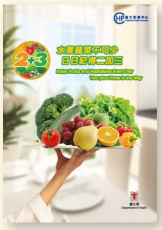

水果蔬菜不可少
Enjoy Fruit and
Vegetables Every Day

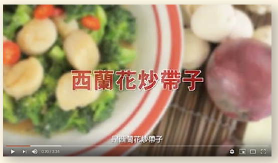

「適飲適食」美饌示範
（適合糖尿病患者）
Cooking Demonstration for
Diabetes-friendly Recipes
(Suitable for Individuals with Diabetes)

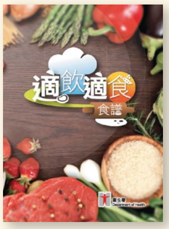

「適飲適食」食譜
（適合糖尿病患者）
Diabetes-friendly Recipes
(Suitable for Individuals with Diabetes)

零酒生活
Live Alcohol Free

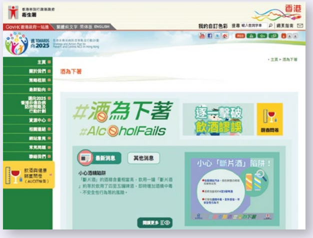

中文

English

酒為下著
Alcohol Fails

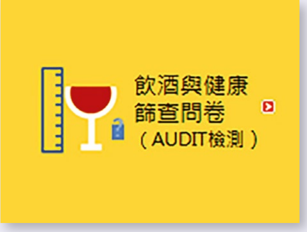

中文

English

飲酒與健康篩查問卷
Alcohol and Health
Questionnaire (AUDIT)

遠離煙草
Stay Away From Tobacco

iOS

Android

戒煙流動應用程式
Quit Smoking Mobile App

2020年修訂 Revised in 2020
政府物流服務署印
