# Supplementary material for: A proactive approach to prevent non-communicable diseases through screening and educating emergency department attendees to adopt healthy lifestyles: Study protocol for a pragmatic, multicenter, randomized controlled trial
Source: PLoS One. 2025 Jul 3;20(7):e0327558. doi: 10.1371/journal.pone.0327558 (PMC12225783; doi:10.1371/journal.pone.0327558)
Supplement: S4 File — (DOCX) [file pone.0327558.s004.docx]

S4 File. Theoretical framework

Theory of planned behaviour

This theory holds that an intention to engage in a health-related behaviour is determined by the proximal factors of attitudes, subjective norms, and perceived behavioural control [1]. Attitudes represent individuals perceived likelihood of performing and appraisal of the consequences of performing a health-promoting behaviour. Subjective norms are the social pressure perceptions to either perform or not perform a health-promoting behaviour. Finally, perceived behavioural control is an individual’s perception of control regarding performing the behaviour.

Foot-in-the-door technique

The foot-in-the-door technique, which was introduced by Freeman and Fraser, emphasises the notion that individuals who initially comply with a small, easy request are more likely to later comply with a larger request [2]. Compliance with the first request or target increases the individual’s confidence and alters their self-perceived capability and willingness regarding further requests or targets. This technique can facilitate the recruitment process and enhance compliance.

Self-determination theory

According to self-determination theory, behavioural regulation is more autonomous when it is internalised, as opposed to being regulated by external factors [3]. Compared with external regulation, autonomous regulation is associated with increased self-efficacy, greater behavioural persistence, longer-term behavioural changes and more positive health behaviour [4]. Autonomy is another influential determinant of behaviour that is emphasised by freedom of choice [3]. There is some evidence that people who have greater autonomy demonstrating greater competence and self-efficacy in achieving behavioural change compared with those with less autonomy [3, 5-6]. As a result, increased autonomy will facilitate a gradual change in risky behaviours.

References:

1. Ajzen I. The theory of planned behavior. Organ Behav Hum. 1991; 50(2): 179-211.
2. Freedman JL, Fraser SC. Compliance without pressure: the foot-in-the-door technique. J Pers Soc Psychol.1966; 4:195.
3. Deci E, Ryan R. Handbook of self-determination research. Rochester, NY: University of Rochester Press, 2002.
4. Li H.C.W., Ho K.Y., Wang M.P., Cheung D.Y.T, Lam K.W.K., Xia W., et al. Effectiveness of a brief self-determination theory-based smoking cessation intervention for smokers at emergency departments in Hong Kong: a randomised controlled trial. JAMA Intern Med 2019 180, 206–14.
5. Bandura A. Self-efficacy: The exercise of control. New York: Freeman, 1997.
6. Williams GC, McGregor HA, Zeldman A, et al. Testing a self-determination theory process model for promoting glycemic control through diabetes self-management. Health Psychology 2004; 23:58-66.
