# Supplementary material for: A proactive approach to prevent non-communicable diseases through screening and educating emergency department attendees to adopt healthy lifestyles: Study protocol for a pragmatic, multicenter, randomized controlled trial
Source: PLoS One. 2025 Jul 3;20(7):e0327558. doi: 10.1371/journal.pone.0327558 (PMC12225783; doi:10.1371/journal.pone.0327558)
Supplement: S5 File — (PDF) [file pone.0327558.s005.pdf]

Demographic Characteristics

Q1. Record the gender

1. Male
2. Female

Q2. What is your age?

________ Years

Q3. What is your highest educational attainment? (Interviewer: read out the answers one by one)

1. Primary or below
2. Lower secondary (S1-S3)
3. Upper secondary (S4-S6) / Matriculation
4. Tertiary (Non-degree, degree or above)
5. Refuse to answer

Q4. What is your marital status? (Interviewer: read out the answers one by one)

1. Never married
2. Married and with child(ren)
3. Married and without child
4. Divorced or Separated
5. Widowed
6. Refuse to answer

Q5a. Are you currently engaged in a job?

1. Yes
2. No (skip to Q5c)

a

Q5b. What is your occupation? (Interviewer: record the details of occupation)

1. Employer/Manager/Administrator
2. Professional
3. Associate Professional
4. Clerk
5. Service worker
6. Shop sales worker
7. Skilled agricultural/fishery worker
8. Craft and related worker
9. Plant and machine operator and assembler
10. Un-skilled worker
11. Other (Please specify : __________)

(skip to Q6)

Q5c. You are a … (Interviewer: read out the answers one by one)

1. Student
2. Homemaker
3. Unemployed person
4. Retired person
5. Others (Please specify________)

(skip to Q7)

Q6. How much is your monthly personal income, including all sources of income?

1. None
2. $1-1,999
3. $2,000-3,999
4. $4,000-5,999
5. $6,000-7,999
6. $8,000-9,999
7. $10,000-11,999
8. $12,000-13,999
9. $14,000-15,999
10. $16,000-17,999
11. $18,000-19,999
12. $20,000-24,999
13. $25,000-29,999

14. $30,000-34,999
15. $35,000-39,999
16. $40,000-44,999
17. $45,000-49,999
18. $50,000 or above
19. Refuse to answer

Q7. How much is your monthly household income, including all sources of income?

1. Less than $2,000
2. $2,000-3,999
3. $4,000-5,999
4. $6,000-7,999
5. $8,000-9,999
6. $10,000-11,999
7. $12,000-13,999
8. $14,000-15,999
9. $16,000-17,999
10. $18,000-19,999
11. $20,000-24,999
12. $25,000-29,999
13. $30,000-34,999
14. $35,000-39,999
15. $40,000-44,999
16. $45,000-49,999
17. $50,000-54,999
18. $55,000-59,999
19. $60,000 or above
20. Don’t know
21. Refuse to answer

Q8. What is the type of your living quarter?

1. Public rental flats
2. Housing Authority subsidised sale flats
3. Housing Society subsidised sale flats
4. Private residential flats
5. Villas/Bungalows/Modern village houses
6. Simple stone structures/Traditional village houses
7. Staff quarters
8. Non-domestic quarters
9. Refuse to answer

END

d

Physical Activity and Leisure-time Exercise

Q1a. During the last 7 days, on how many days did you do vigorous physical activities? Vigorous physical activities are those that make you breathe much harder and your heart beat much faster than normal, e.g., running, aerobics, football, swimming, heavy physical work, jogging, etc., and you did these activities for at least 10 minutes at a time.

__________ Days

Q1b. [Only ask those whose answers in Q1a are greater than or equal to "1"]
On those days that you have performed vigorous physical activities for at least 10 minutes, how much time on average per day did you usually spend doing vigorous physical activities?

_________ Minutes

Q2a. During the last 7 days, on how many days did you do moderate physical activities? Moderate physical activities are those that make you breathe somewhat harder and your heart beat somewhat faster than normal, e.g., bicycling, washing cars/polishing, fast walking, cleaning windows, etc. and you did these activities for at least 10 minutes at a time.

__________ Days

Q2b. [Only ask those whose answers in Q2a are greater than or equal to "1"]
On those days that you have performed moderate physical activities for at least 10 minutes, how much time on average per day did you usually spend doing moderate physical activities?

___________ Minutes

Q3a. During the last 7 days, on how many days did you walk for at least 10 minutes at a time? This includes walking to offices/schools, walking to travel from place to place, and walking for leisure.

_____________ Days

Q3b. [Only ask those whose answers in Q3a are greater than or equal to "1"]
On those days that you have walked for at least 10 minutes at a time, how much time on average did you usually spend walking in one of those days?

__________ Hours ___________ Minutes

Q4. During the last 7 days, how much time on average did you usually spend sitting on a weekday? This includes time spent sitting at work, at home or other places, visiting friends, traveling on public transport, reading and lying down to watch television. (Interviewer's prompts: If the respondent cannot answer the daily average time, then say: Please try to make an estimate as accurate as possible.)

_________Hours __________ Minutes

Q5. During the past 30 days, how often did you exercise in your leisure time, which at least made you breathe somewhat harder than normal and sweat?

1. Once or more a day
2. 4-6 times/week
3. 2-3 times/week
4. Once a week
5. 2-3 times a month
6. Once a month
7. Less than once a month

Fruit and Vegetable Consumption

Q1a. On average, how many days do you eat fruit each week? (excluding fruit juice)

1. 1 Day
2. 2 Days
3. 3 Days
4. 4 Days
5. 5 Days
6. 6 Days
7. 7 Days
8. None (skip to Q2a)

Q1b. On average, how many fruit did you eat on one of those days?
(Interviewer's prompts: One fruit equals to 1 medium-sized apple or orange, 1 medium sized banana, or 2 kiwi fruits or plums, or half bowl of small fruits like grapes or strawberries. Ask exactly what they ate and then convert using table. The numbers can be recorded as half such as 0.5 or 1.5.)

_____ Pieces

Q2a. On average, how many days do you eat vegetables each week? (excluding vegetable juice)

1. 1 Day
2. 2 Days
3. 3 Days
4. 4 Days
5. 5 Days
6. 6 Days
7. 7 Days
8. None (skip to Q3)

Q2b. On average, how many bowls of cooked vegetables did you eat on one of those days?
(Interviewer's prompts: One bowl refers to the size of a rice bowl. The numbers can be recorded as half such as 0.5 or 1.5. For uncooked leafy vegetables, half the total.)
________ Bowls

Q3. On average, how many days in the week do you drink at least one cup of fruit or vegetable juice? "Juice" refers to freshly squeezed juice or those that are labelled 100% or pure fruit/vegetable juice. A cup means 250 ml in volume or a standard-sized tetra pack of juice drink.

1. 1 Day
2. 2 Days
3. 3 Days
4. 4 Days
5. 5 Days
6. 6 Days
7. 7 Days
8. None

Meat Consumption

Q1a. In the past 30 days, how many days on average did you eat red meat each week? Common examples of red meat include pork, beef, and lamb.

1. Daily
2. 6 days per week
3. 5 days per week
4. 4 days per week
5. 3 days per week
6. 2 days per week
7. 1 day per week
8. Less than 1 day per week

Q1b. [Only ask those who answers "1" to "7" in Q1a]
Taking only the days you had eaten red meat into account, on average, how many taels / slices of red meat about the size of a mah-jong tile did you eat in one day? (Interviewer's prompts: A tael of meat also equates to 40 grams, 1.33 ounces or 4 slices, and 1 pound is equivalent to 12 taels. The numbers can be recorded as half such as 0.5 or 1.5 taels.)

_______ Taels

Q2a. In the past 30 days, how many days on average did you eat white meat each week? Common examples of white meat include poultry and fish.

1. Daily
2. 6 days per week
3. 5 days per week
4. 4 days per week
5. 3 days per week
6. 2 days per week
7. 1 day per week
8. Less than 1 day per week

Q2b. [Ask those who answers "1" to "7" in Q2a]
Taking only the days you had eaten white meat into account, on average, how many taels / slices of white meat about the size of a mahjong tile did you eat in one day? (Interviewer's prompts: A tael of meat also equates to 40 grams, 1.33 ounces or 4 slices, and 1 pound is equivalent to 12 taels. The numbers can be recorded as half such as 0.5 or 1.5 taels.)

_______ Taels

Q3. In the past 30 days, how many days on average did you eat processed meat each week? They include canned meat, cured meat or smoked meat, such as luncheon meat, ham, sausages, bacon and Chinese preserved meat.

1. Daily
2. 6 days per week
3. 5 days per week
4. 4 days per week
5. 3 days per week
6. 2 days per week
7. 1 day per week
8. Less than 1 day per week

Smoking Pattern

Q1. Have you ever smoked before? (Interviewer: read out the answers one by one)

1. Yes, but not now
2. Yes, and still smoking (skip to Q3a)
3. Never (skip to next section)

Q2. How long have you stopped smoking? (Interviewer: read out the answers one by one)

1. Less than 1 month (skip to next section)
2. 1 month to 1 year (skip to next section)
3. More than 1 year (skip to next section)

Q3a. How many cigarettes do you smoke on average per day? (Interviewer: Do not read out the answers)

1. Less than 1 per day
2. 1-10 per day
3. 11-20 per day
4. More than 20 per day

Q3b. In the next 6 months, are you planning to quit smoking or smoke less?

1. Yes, plan to quit smoking
2. Yes, plan to smoke less
3. No plan to change
4. Not sure/Don't know

Pattern of Alcohol Consumption

Q1a. During the past year, did you have a drink containing alcohol?

1. Yes (skip to Q2a)
2. No

Q1b. In the next 6 months, will you have a drink containing alcohol?

1. Yes (skip to next section)
2. No (skip to next section)
3. Not sure/Don't know (skip to next section)

Q2a. How often do you have a drink containing alcohol?

1. Less than monthly
2. Once a month
3. 2 to 3 times a month
4. Once a week
5. 2 times a week
6. 3 times a week
7. 4 times a week
8. 5 times a week
9. 6 times a week
10. Once a day

Q2b. On a typical day when you are drinking, what type of drinks containing alcohol and how much do you have?

(Interviewer: please make reference of the following pictures for calculating the number of drinks appropriately. One unit equals to 10 grams of ethanol.)

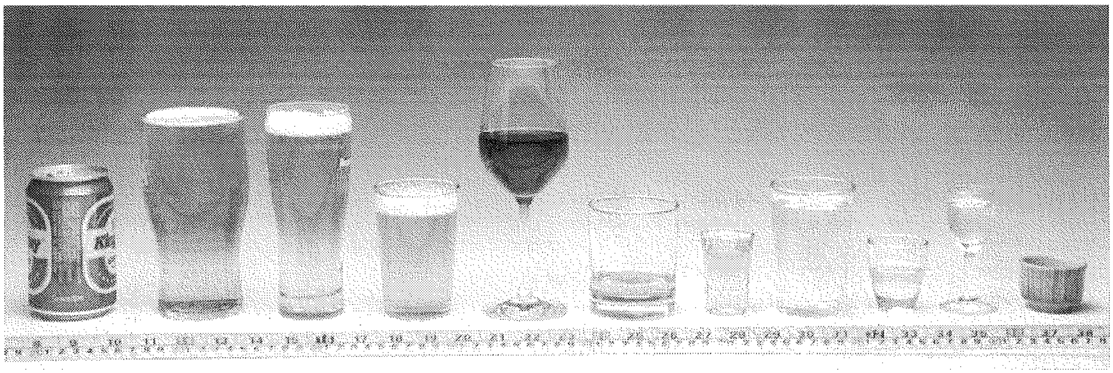

Beer				Red/White wine	Spirit		Rice wine		Shorgum	Sake
1 can ~330 ml	1 pint ~568 ml	Half pint ~284 ml	1 glass ~180 ml	1 glass ~125 ml	1 peg ~40-50 ml	1shot ~22 ml	1 glass ~180 ml	1 small glass ~ 20 ml	1 small glass ~20 ml	1 small glass ~20ml
( ) can/	( ) glass	( ) glass	( ) glass	( ) glass	( ) glass	( ) glass	( ) glass	( ) glass	( ) glass	( ) glass
1.3 units	2.2 units	1.1 units	0.7 units	1.2 units	1.3 units	0.7 units	5.7 units	0.6 units	0.8 units	0.3 units

_______ Units of drinks

Q2c. How often do you have at least 5 or more drinks on one occasion? That means the total number of glasses and cans of any type of alcohol, and one occasion means period of a few hours.

1. Never
2. Less than monthly
3. Once a month
4. 2 times a month
5. 3 times a month
6. Once a week
7. 2-3 times a week
8. 4-6 times a week
9. Daily or almost daily

Q2d. In the next 6 months, are you planning to change your drinking habit?

1. Yes, plan to drink more
2. Yes, plan to drink less
3. Yes, plan to stop drinking completely
4. No plan to change
5. Not sure/Don't know

人口特徵

Q1. 記錄性別

1. 男
2. 女

Q2. 請問你多少歲？

________歲

Q3. 請問你最高的教育程度是？[訪問員：請讀出個別答案]

1. 小學或以下
2. 初中 (中一至中三)
3. 高中 (中四至中六) ／預科
4. 專上教育 (非學位、學位或以上)
5. 拒絕回答

Q4. 請問你的婚姻狀況是？ [訪問員：請讀出個別答案]

1. 未婚
2. 已婚並有孩子
3. 已婚但沒有孩子
4. 分居或離婚
5. 喪偶
6. 拒絕回答

Q5a. 你現時有工作嗎？

1. 有
2. 沒有 (跳答至Q5c)

Q5b. 你的職業是什麼呢？ [訪問員：請記錄詳細職業資料]

1. 僱主／經理／行政人員
2. 專業人員
3. 輔助專業人員
4. 文員
5. 服務工作人員
6. 商店銷售人員
7. 漁農業熟練工人
8. 工藝及有關人
9. 機台及機器操作員及裝配員
10. 非技術工人
11. 其它: (請說明_______)

(1–11 跳答至 Q6)

Q5c. 你是一位 …… 。 [訪問員：請讀出個別答案]

1. 學生
2. 家庭主婦
3. 失業／待業人士
4. 退休人士
5. 其它 (請說明_______)

(1–5 跳答至 Q7)

Q6. 包括所有入息來源，你的每月個人總收入是？

1. 沒有收入
2. $1-1,999
3. $2,000-3,999
4. $4,000-5,999
5. $6,000-7,999
6. $8,000-9,999
7. $10,000-11,999
8. $12,000-13,999
9. $14,000-15,999
10. $16,000-17,999
11. $18,000-19,999
12. $20,000-24,999

13. $25,000-29,999
14. $30,000-34,999
15. $35,000-39,999
16. $40,000-44,999
17. $45,000-49,999
18. $50,000 或以上
19. 拒絕回答

Q7. 包括所有入息來源，你的每月家庭總收入是？

1. $2,000以下
2. $2,000-3,999
3. $4,000-5,999
4. $6,000-7,999
5. $8,000-9,999
6. $10,000-11,999
7. $12,000-13,999
8. $14,000-15,999
9. $16,000-17,999
10. $18,000-19,999
11. $20,000-24,999
12. $25,000-29,999
13. $30,000-34,999
14. $35,000-39,999
15. $40,000-44,999
16. $45,000-49,999
17. $50,000-54,999
18. $55,000-59,999
19. $60,000 或以上
20. 不知道
21. 拒絕回答

C

Q8. 請問你現正居住的房屋類型是 ？

1. 公營租住單位
2. 房屋委員會資助出售單位
3. 房屋協會資助出售單位
4. 私人住宅單位
5. 別墅／平房／新型村屋
6. 簡單磚石蓋搭建築物／傳統村屋
7. 員工宿舍
8. 非住宅用屋宇單位
9. 拒絕回答

完

d

體能活動及餘暇時間做的運動

Q1a. 在過去的七天，你有多少天有做劇烈強度的體能活動呢？劇烈強度的體能活動是指會令你呼吸及心跳比平常快很多的體能活動，例如: 跑步、跳健康舞、踢足球、游泳、做粗重工作、緩步跑等，而你每一次最少會做10分鐘或以上的。

__________天

Q1b. [只供Q1a答案為大過或等於"1"的被訪者回答]
在你有做劇烈強度體能活動的日子裡，只計算每次至少做10分鐘或以上的，你平均一天會用多少時間做劇烈強度的體力活動呢？

_________分鐘

Q2a. 在過去的七天，你有多少天有做中等強度的體能活動呢？中等強度的體能活動是指會令你呼吸及心跳比平常快一些的體能活動，例如：踏單車，洗車打臘，快步走，抹窗等，而你每一次最少會做 10分鐘或以上的。

__________天

Q2b. [只供Q2a答案為大過或等於"1"的被訪者回答]
在你有做中等強度體能活動的日子裡，只計算每次做至少10分鐘或以上的，你平均一天會用多少時間做中等強度的體能活動呢？

___________分鐘

Q3a. 在過去的七天，你有多少天有步行至少 10 分鐘或以上的？包括步行上／上學，由一個地方步行到另一個地方，和日常散步等。

_____________天

Q3b. [只供Q3a答案為大過或等於“1”的被訪者回答]
只計算每次 10 分鐘或以上的步行，你平均一天用多少時間步行呢？

__________小時 ___________分鐘

Q4. 在過去的七天，只計算星期一到星期五，你平均一天有多少時間坐著呢？這包括坐在辦公室、家裡或任何地方、拜訪朋友坐著的時候、坐著乘搭公共交通工具、坐著看書或躺下看電視。[訪問員: 如果被訪者不能夠回答每天的平均時間，請讀出：請盡量準確地估計一下。]

_________小時 __________分鐘

Q5. 在過去的三十天，你有幾經常在餘暇時間做運動，而該運動至少令你呼吸比平常急速和流汗？

1. 每日 1 次或以上
2. 每星期 4-6 次
3. 每星期 2-3 次
4. 每星期 1 次
5. 每月 2-3 次
6. 每月 1 次
7. 少於每月 1 次

進食水果及蔬菜

Q1a. 你平均一個星期有多少天會吃水果？ (不包括果汁)

1. 1 天
2. 2 天
3. 3 天
4. 4 天
5. 5 天
6. 6 天
7. 7 天
8. 沒有吃 (跳答至Q2a)

Q1b. 只計算你有吃水果的日子, 你平均一天會吃多少個水果呢？)
(訪問員： 1 個水果等於 1 個中等大小的蘋果或橙，1 隻中等大小的香蕉，或 2 個奇異果或李子，或半碗葡萄或草莓 。請追問他們吃什麼水果，然後用表轉換。數字可被記錄如 0.5 或 1.5)

_____ 個

Q2a. 你平均一個星期有多少天會吃瓜菜？ (不包括蔬菜汁)

1. 1 天
2. 2 天
3. 3 天
4. 4 天
5. 5 天
6. 6 天
7. 7 天
8. 沒有吃 (跳答至Q3)

Q2b. 只計算你有吃瓜菜的日子在內，你平均一天會吃多少碗份量相等於一個飯碗煮熟的瓜菜呢？ (訪問員：可記半碗如 0.5 碗或 1.5 碗; 如未經烹調的葉菜，須減半。)

________碗

Q3. 你平均一個星期有多少天會喝至少一杯果汁或蔬菜汁？果汁或蔬菜汁是指鮮榨、標籤 100%或純果汁或純蔬菜汁。一杯的份量約為 250 毫升或相等於普通盒裝飲料的份量。

1. 1 天
2. 2 天
3. 3 天
4. 4 天
5. 5 天
6. 6 天
7. 7 天
8. 沒有喝

進食肉類

Q1a. 在過去的三十天，你平均一個星期有幾多日食 紅肉？ 紅肉常見例子包括豬肉、牛肉和羊肉。

1. 每日
2. 每星期 6 日
3. 每星期 5 日
4. 每星期 4 日
5. 每星期 3 日
6. 每星期 2 日
7. 每星期 1 日
8. 少於每星期 1 日

Q1b. [只問那些在 Q1a 回答 "1" 至 "7" 的被訪者]

只計算你有食紅肉的日子，請問你平均一日食多少兩紅肉呢？ 一兩肉約相等於一隻打牌用的麻雀般大小的肉塊。 (提示訪問員：一兩肉亦相等於 40 克、1.33 安士或約 4 片肉，另外一磅相等於 12 兩。可記錄半塊的份量如 0.5 兩或 1.5 兩)

_______兩

Q2a. 在過去的三十天，你平均一個星期有幾多日食 白肉？ 白肉常見例子包括家禽和魚。

1. 每日
2. 每星期 6 日
3. 每星期 5 日
4. 每星期 4 日
5. 每星期 3 日
6. 每星期 2 日
7. 每星期 1 日
8. 少於每星期 1 日

Q2b. [只問那些在 Q2a 回答 "1" 至 "7" 的被訪者]

只計算你有食白肉的日子，請問你平均一日食多少兩白肉呢？ 一兩肉約相等於一隻打牌用的麻雀般大小的肉塊。(訪問員：一兩肉亦相等於 40 克、1.33 安士或約 4 片肉，另外一磅相等於 12 兩。可記錄半塊的份量如 0.5 兩或 1.5 兩)

_______兩

Q3. 在過去的三十天，你平均一個星期有幾多日食 經過加工處理的肉 ？這包括罐裝肉、醃製或煙燻過的肉，例如午餐肉、火腿、香腸、煙肉及臘肉。

1. 每日
2. 每星期 6 日
3. 每星期 5 日
4. 每星期 4 日
5. 每星期 3 日
6. 每星期 2 日
7. 每星期 1 日
8. 少於每星期 1 日

抽煙情況

Q1. 你有沒有曾經抽煙？[訪問員：請續一讀出答案]

1. 有，但已戒掉
2. 有，現在還有抽煙（跳答至Q3a）
3. 從來沒有（跳答至下部分）

Q2. 你戒掉了多久？[訪問員：請續一讀出答案]

1. 少於1個月 (跳答至下部分）
2. 1個月至1年（跳答至下部分）
3. 超過1年（跳答至下部分）

Q3a. 你平均一天會抽多少枝香煙呢？[訪問員：不可讀出答案]

1. 每日少過1枝
2. 每日1-10枝
3. 每日11-20枝
4. 每日超過20枝

Q3b. 於未來的六個月，你有沒有打算戒煙或減少吸煙？

1. 有，打算戒煙
2. 有，打算減少吸煙
3. 無打算改變
4. 不肯定／不知道

飲酒習慣

Q1a. 在過去的一年，你有否飲含酒精的飲品？

1. 有 （跳答至 Q2a）
2. 沒有

Q1b. 於未來的六個月，你會否飲酒？

1. 會 （跳答至下部分）
2. 不會 （跳答至下部分）
3. 不肯定／不知道（跳答至下部分）

Q2a. 你有幾經常飲酒？

1. 每月少於一次
2. 每月一次
3. 每月二至三次
4. 每星期一次
5. 每星期二次
6. 每星期三次
7. 每星期四次
8. 每星期五次
9. 每星期六次
10. 每日一次

Q2b. 在你飲酒的日子裡，通常會飲用那類的酒精飲品和一日會飲用多少？

[訪問員：請參閱下圖其他例子，並計算飲用的單位數量。以 1 單位含 10 克酒精計算。]

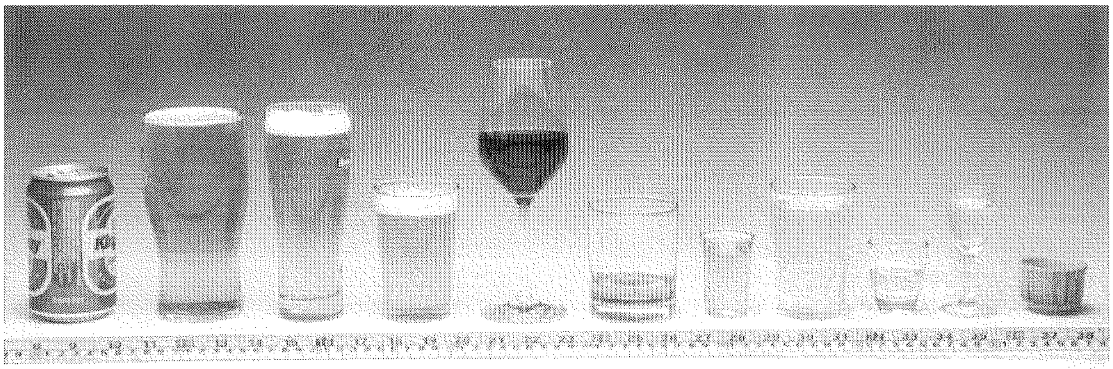

啤酒				紅酒/白酒	烈酒		米酒		茅台酒	日本清酒
1 罐<br>~330<br>毫升	1 品脫<br>~568<br>毫升	半品脫<br>~284<br>毫升	1 水杯<br>~180<br>毫升	1 杯<br>~125<br>毫升	1 peg<br>~40-50<br>毫升	1 杯<br>~22<br>毫升	1 水杯<br>~180<br>毫升	1 小杯<br>~ 20<br>毫升	1 小杯<br>~20<br>毫升	1 小杯<br>~20<br>毫升
( )<br>罐	( )<br>杯	( )<br>杯	( )<br>杯	( )<br>杯	( )<br>杯	( )<br>杯	( )<br>杯	( )<br>杯	( )<br>杯	( )<br>杯
1.3<br>單位	2.2<br>單位	1.1<br>單位	0.7<br>單位	1.2<br>單位	1.3<br>單位	0.7<br>單位	5.7<br>單位	0.6<br>單位	0.8<br>單位	0.3<br>單位

_______單位

Q2c. 你有幾經常一次過飲至少5罐或5杯酒精飲品？這是指任何類型的酒杯或罐的總數，而一次過是指在幾個小時之內。

1. 從不
2. 每月少於一次
3. 每月一次
4. 每月二次
5. 每月三次
6. 每星期一次
7. 每星期二至三次
8. 每星期四至六次
9. 每日或幾乎每日

Q2d. 於未來的六個月，你有無打算改變你的飲酒習慣？

1. 有，打算增加飲酒
2. 有，打算減少飲酒
3. 有，打算完全停止飲酒
4. 無打算改變
5. 不肯定／不知道
