# Supplementary material for: A proactive approach to prevent non-communicable diseases through screening and educating emergency department attendees to adopt healthy lifestyles: Study protocol for a pragmatic, multicenter, randomized controlled trial
Source: PLoS One. 2025 Jul 3;20(7):e0327558. doi: 10.1371/journal.pone.0327558 (PMC12225783; doi:10.1371/journal.pone.0327558)
Supplement: S7 File — (DOCX) [file pone.0327558.s007.docx]

S7 File. Cost-effectiveness analysis (CEA)

A health economist will conduct a cost-effectiveness analysis (CEA) in accordance with recommendations of the Agency for Health Care Policy and Research [1] and the Health and Medicine of the US Public Health Service [2]. Utility scores based on EQ-5D-5L assessed at baseline, 6 and 12 months will be integrated over the 12-month study period using a trapezoidal approximation to calculate quality-adjusted life-years (QALYs) for the participants. The CEA will be populated using empirical 12-month RCT data. An ingredients approach will be used to estimate the cost of the intervention programme, including intervention material (e.g., leaflets and videos), the administration cost, and the time taken to deliver the intervention. We will report the incremental cost-effectiveness ratio (ICER) in terms of incremental cost per QALY gained by the intervention as follows: (cost of intervention − cost of control) / (QALY of intervention − QALY of control).38 Biased-corrected bootstrapping will be used to generate 20,000 pairs of incremental costs and QALYs and plot them on a cost-effectiveness plane to graphically illustrate the uncertainty surrounding the cost-effectiveness ratio. A cost- effectiveness acceptance curve will be generated to demonstrate the probability of cost- effectiveness of the intervention at different thresholds of willingness-to-pay for a QALY gained. The intervention is regarded as cost-effective over control if the ICER is less than 1 to 3 times the gross domestic product per capita, as indicated by the World Health Organization [3] and cost- effectiveness threshold derived by opportunity cost in Hong Kong [4]. All cost data will be valued based on non-subsidised costs on the study starting date in terms of Hong Kong dollar.

References:

1. Cromwell J, Bartosch WJ, Fiore MC, Hasselblad V, Baker T. Cost-effectiveness of the clinical practice recommendations in the AHCPR guideline for smoking cessation. JAMA. 1997;278:1759-66.

2. Weinstein MC, Siegel JE, Gold MR, Kamlet MS, Russell LB. Recommendation of the panel of cost-effectiveness in health and medicine. JAMA.1996;276(15):1253-1258.

3. Marseille E, Larson B, Kazi DS, Kahn JG, Rosen S. Thresholds for the cost-effectiveness of interventions: alternative approaches. Bull World Health Organ. 2015;93(2):118-124.

4. Woods B, Revill P, Sculpher M, Claxton K. Country-level cost-effectiveness thresholds: initial estimates and the need for further research. Value Health. 2016;19(8):929-935.
